# Tips and tricks in triple-negative breast cancer: how to manage patients in real-life practice?

**DOI:** 10.3332/ecancer.2011.217

**Published:** 2011-07-19

**Authors:** M Piccart, G Viale, P Ellis, M Abramowicz, L Carey

**Affiliations:** 1 Free University of Brussels, Brussels, Belgium; 2 Department of Medicine, Jules Bordet Institute, Brussels, Belgium; 3 University of Milan School of Medicine, Milan, Italy; 4 Division of Pathology and Laboratory Medicine, European Institute of Oncology, Milan, Italy; 5 Guy’s Hospital, London, UK; 6 Cancer Medicine, King’s College, London, UK; 7 Department of Genetics, Centre of Human Genetics, Hôpital Erasme, Brussels, Belgium; 8 Human and Medical Genetics, Free University of Brussels, Belgium; 9 Department of Medicine, Division of Hematology/Oncology, University of North Carolina, Chapel Hill, North Carolina, USA

## Introduction

This article has been developed following, and drawing on the content of, a satellite meeting at the fifth International Breast Cancer Conference, held in Paris, France, on 29 January 2011. The purpose of the meeting was to examine several questions relating to triple-negative breast cancer (TNBC):

- How should TNBC be defined?
- Are there clinically important TNBC subtypes?
- Should patients be given adjuvant or neoadjuvant treatment for TNBC?
- Should patients with TNBC and their families have genetic tests?
- How should relapsing or metastatic TNBC be treated?

Using a real-life case study, at each stage of the patient care pathway from diagnosis through assessment to treatment, the audience was encouraged to vote on potential decisions, before an expert panel on which we all sat discussed the evidence and presented what we consider constitutes best clinical practice.

In this article we share the proceedings of the meeting, which we believe contained valuable educational content of potential interest to the wider healthcare community.

**Case study**

Carole is a 37-year-old primary school teacher, with a husband and two young children (a 7-year-old daughter and a 3-year-old son). She presented, in January 2010, with a palpable large tumour mass (6 cm) in her right breast. She was diagnosed with a right mammary tumour (T4N2), with carcinomatous mastitis and four suspicious lymph nodes. Biopsy confirmed a grade 3 invasive ductal carcinoma that was negative for oestrogen receptors (ER), progesterone receptors (PgR) and human epidermal growth factor receptor 2 (HER2).

## How should TNBC be defined?

### Audience voting

When offered potential definitions for ER or PgR negativity, most of the audience (58%) agreed on a cutoff of 1% positive cells, although around a third would use 10% as a cutoff. Most participants (89%) would use fluorescence *in situ* hybridization (FISH) to confirm HER2 status if the immunohistochemistry (IHC) test was 2+; in some countries (such as Belgium), FISH testing is mandatory for trastuzumab reimbursement, regardless of IHC score.

### Expert opinion of Professor Giuseppe Viale

Concordance on thresholds for defining ER and PgR negativity is vital to ensure that pathologists and clinicians describe and treat patients consistently. The joint American Society of Clinical Oncology (ASCO) and College of American Pathologists guidelines on IHC testing in breast cancer [1] have specified a cutoff of 1% immunoreactive cells.

There is a concern that patients with 1–10% immunoreactive cells are not responsive to endocrine therapy; however, review of patients recruited into clinical trials of tamoxifen and aromatase inhibitors has shown that these patients have a greater benefit from endocrine treatment than those who have less than 1% immunoreactive cells. The question should not be “Is it worth treating this patient?” but rather “Am I sure I can deny this patient the possible benefit of endocrine treatment?”

Pathologists need to confirm the lack of immunoreactive cells (negative ER status) in the tissue sample by a positive control in normal ducts to avoid any false negative reports and to be assured of the sensitivity and specificity of the assay. Evidence shows that there is much room for improvement in accuracy, with up to 20% false-negative results for ER/PgR, more than 12% false-positive results for PgR, and up to 15% false-positive results for HER2 [2].

## Are there clinically important TNBC subtypes?

### Audience voting

Most participants would not request additional tumour analyses (e.g. epidermal growth factor receptor (EGFR), cytokeratin 5/6, 14 and 17). The audience was divided almost equally over the issue of histological subtyping for patients who have been classified as having TNBC.

### Expert opinion of Professor Giuseppe Viale

Patients with TNBC have some typical features, as shown in Table 1 [3,4], but there is a great heterogeneity in the underlying pathological tumour type (Table 2). The vast majority of tumours (70–85% [5,6]) are invasive ductal carcinomas not otherwise specified, but there are many other TNBC tumour subtypes and prognosis varies greatly. The answer to the question “Are you interested in subtyping TNBC?” should be “yes!”

Around 80% of TNBC has a basal-like gene expression [7]. Basal-like breast cancer is defined by expression of around 500 different messenger RNA molecules. At the messenger RNA level, such tumours have a relatively high expression of a number of markers, including cytokeratin 5 and 17, EGFR, KIT, laminin, collagen type XVII, calponin 1 and calveolin 2, and a relatively low expression of ER or HER2. Currently, assessment of these markers has no impact on clinical treatment decisions, although the potential prognostic implications of surrogate markers is being investigated.

When confronted with an apparently triple-negative tumour, pathologists should follow a hierarchical approach to assessment, focusing first on morphology to identify the subtypes with a good prognosis (i.e. adenoid-cystic, medullary, metaplastic low-grade, apocrine low-grade). They should then confirm that the immunophenotype is truly nonendocrine-responsive (with <1% ER/PgR immunoreactive cells, and no false-negative results) and reassess equivocal IHC results. Further phenotyping could be performed for investigational purposes.

In summary, the definition of TNBC needs to be standardized and agreed by the international community. Clinicians need to understand the differences between TNBC and basal-like breast cancer and the importance of using a hierarchical approach to diagnosis, focusing on thorough evaluation of morphological features, followed by accurate assessment of receptor status (ER, PgR and HER2), with use of surrogate IHC markers or gene expression profiling assays to identify basal-like carcinomas if deemed appropriate.

## Should patients be given adjuvant or neoadjuvant chemotherapy for TNBC?

### Audience voting

Given a choice of potential treatments for Carole, the patient in this case study, 40% of the audience opted for neoadjuvant chemotherapy alone and 38% would consider additional adjuvant chemotherapy depending on pathological response to neoadjuvant treatment. The most popular selection of neoadjuvant chemotherapy was a sequential regimen of anthracycline followed by taxane (48%) or combination of these agents (38%). Around 13% of participants would consider neoadjuvant platinum-based treatment.

**Case study**

In January 2010, the patient was started on a dose-dense epirubicin + cyclophosphamide regimen followed by weekly paclitaxel 80 mg/m^2^. After four cycles of the anthracycline treatment, there was evidence of a slight decrease in tumour size but lymph nodes were still palpable. After 12 weeks of paclitaxel, the patient had clinical and radiological improvement (no lymph nodes, no measurable disease). After tumorectomy and axillary lymph-node dissection in June 2010, the patient had no residual tumour and no lymph-node involvement (pT0N0).

### Expert opinion of Professor Paul Ellis

**Figure 1:**
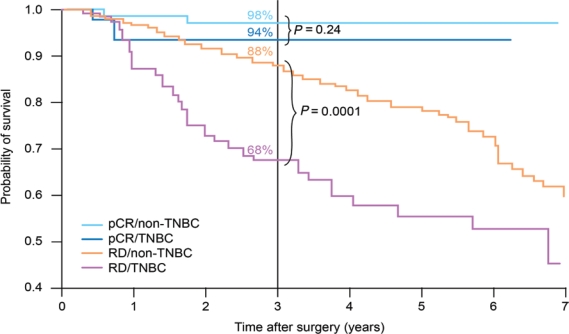
Survival by tumour type and response status (adapted from ref. [10]). pCR, pathological complete response; TNBC, triple-negative breast cancer; RD, residual disease.

Using pathological complete response (pCR) as a surrogate endpoint, there is evidence that TNBC is a chemo-responsive disease, with pCR rates of 20–45% after anthracycline or anthracycline/taxane-based treatments [8–11]. These rates are similar to those achieved in women with HER2^+^ disease, and substantially better than in endocrine-responsive disease. However, as Figure 1 shows, although patients with TNBC who achieve a pCR have a good prognosis, those without a pCR have a poor outcome, with a higher risk of relapse [10].

Neoadjuvant therapy may not have a role in all patients (e.g. those with small tumours that can be treated with surgery and standard adjuvant chemotherapy) but its use in many patients makes sense—in particular those in whom breast conservation is not possible or who have clinically involved nodes. In clinical trials, neoadjuvant therapy helps to address questions about treatment choices—an example would be the use of different chemotherapy backbones to support novel therapeutic approaches such as inhibitors of poly-(ADP) ribose polymerase (PARP)—and to use translational research to identify subgroups of TNBC patients who might benefit from different treatments.

Anthracyclines are commonly used in TNBC, and there is clinical trial evidence of a survival benefit versus no treatment (hazard ratios ranging from 0.35 (95% confidence interval 0.18–0.68) for nonbasal subtypes to 0.54 (0.27–1.08) for basal subtypes [12].) Furthermore, compared with cyclophosphamide + methotrexate + 5-fluorouracil, patients with TNBC achieved a superior benefit from anthracyclines [13], although the MA5 study [14] found the opposite result in a relatively small group of patients receiving cyclophosphamide + methotrexate + 5-fluorouracil or cyclophosphamide + epirubicin + 5-fluorouracil.

**Figure 2:**
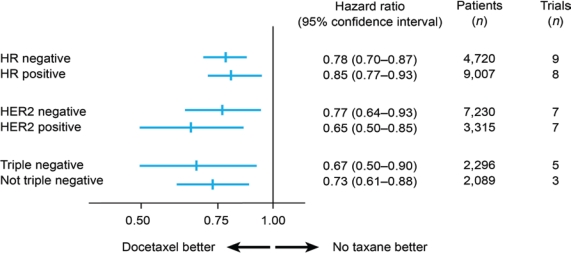
Disease-free survival with docetaxel by breast cancer subtype (adapted from ref. [15])

A meta-analysis of randomized trials has shown docetaxel to be as effective in TNBC as in non-TNBC patients in terms of disease-free survival (Figure 2) [15]: the hazard ratios for docetaxel versus no docetaxel were 0.67 (95% confidence interval 0.50–0.90) in 2,296

TNBC patients (five studies) and 0.73 (0.61–0.88) in 2,089 non-TNBC patients (three studies). A similar benefit has been confirmed for paclitaxel [16]. Therefore, it seems reasonable that full-dose anthracycline/taxane-based therapy should be the standard of care for TNBC patients.

Data for platinum agents are less mature, although a number of studies in the neoadjuvant setting suggest a benefit in terms of pCR rates, particularly in patients with *BRCA* mutations (72% pCR with cisplatin [17]). However, in less selected TNBC patients, the pCR rates are only around 15–30% with cisplatin [18,19] and 22–40% with carboplatin [19–22]. A number of questions about platinum agents remain to be answered, such as the choice of agent and the relative benefit versus nonplatinum chemotherapies. For the time being, although they show promise, they should not yet be considered the standard of care in the neoadjuvant or adjuvant setting.

The potential impact of targeted therapies is being explored in TNBC. Evidence suggests that the addition of bevacizumab to an anthracycline/taxane combination may be beneficial in ER-negative patients, although there is no benefit in the whole breast cancer population [23]. The addition of bevacizumab to chemotherapy is also being explored in the BEATRICE study [24]. A range of PARP inhibitor studies, being developed by different cooperative groups, could provide information on ways to optimize chemotherapy in the neoadjuvant treatment of TNBC.

In summary, the evidence supports anthracycline/taxane combination therapy for early TNBC. The patient should receive a full course of treatment, whether in the neoadjuvant or the adjuvant setting. If she has received the full course before surgery, there is no need for further adjuvant chemotherapy outside a clinical trial. Our challenge is to help those patients who we know will do badly if they do not achieve a pCR, ideally exploring the use of new therapies with minimal use of cytotoxic agents.

## Should patients and their families have genetic tests?

**Case study**

The familial history should be assessed in all young women with breast cancer. In this case study, Carole’s mother was diagnosed with hormone-sensitive breast cancer at the age of 56 years but there is no other confirmed case in the maternal family.

### Audience voting

Two thirds of the audience would look for a genetic mutation (specifically *BRCA1* and possibly *BRCA2*) in this patient. Assuming a genetic mutation was found, around a quarter of participants would screen the patient’s sisters as well, although very few would screen further family members.

### Expert opinion of Professor Marc Abramowicz

Only a small minority of breast cancers are due to a hereditary mutation in a single gene (perhaps 5% [25]). Inherited mutations usually involve the *BRCA1* or *BRCA2* gene. Some families, however, have other mutations, which may not always be easy to identify with existing techniques.

When deciding whether to test a woman for a hereditary mutation, it must be borne in mind that other members of the family will be affected too, although it may not be feasible to test everyone at first. Furthermore, the result of the test does not guarantee that breast cancer will or will not develop. A sister without the mutation may still have breast cancer by chance, whereas many women with mutations do not develop the disease [26]. Nonetheless, women can be assigned to risk categories to determine appropriate risk-reducing and management strategies.

Only a minority of TNBC patients are *BRCA1* carriers, even in conspicuous familial cases when both the mother and daughter had onset in their early 30s [27]. As a result, it would be inappropriate to assess all TNBC patients for *BRCA1* mutations: such testing would be labour-intensive and expensive, and would result in too many false-negatives and false-positives (i.e. genetic variants that do not result in disease), with associated mistaken reassurances or psychological impact on family members. Therefore, ASCO recommends that genetic testing should be performed in selected patients with personal or family history features suggesting a genetic cancer susceptibility (Table 3), with appropriate genetic counselling [28]. The test needs to be adequately interpreted and must be able to provide results that can guide diagnosis or treatment decisions for the patient or family members.

For Carole, in this case study, her father’s family history should be reviewed too, as men can also transmit mutations. As she had TNBC before the age of 50 years, she should be considered for *BRCA1* testing. If she is found to have a *BRCA1* mutation, her sisters should be offered testing, as should her daughter when she is an adult (i.e. around 20 years old). Her mother should also be tested, even though she has a history of ER-positive disease.

## How should recurring or metastatic TNBC be treated?

**Case study**

Despite having achieved a pCR in June, Carole experienced a rapid cutaneous relapse and neuropathic pain. On clinical examination in October 2010, she was found to have skin infiltration and a right axillary mass. Imaging showed right diffuse carcinomatous mastitis, and there was evidence of axillary and retroperitoneal para-aortic lymph-node involvement.

### Audience voting

More than half of the audience suggested entering her into a clinical trial of a PARP inhibitor. Other options included platinum-based or taxane-based (docetaxel + capecitabine or gemcitabine; paclitaxel + bevacizumab) chemotherapy.

### Expert opinion of Professor Lisa Carey

The heterogeneity described earlier for TNBC continues to manifest when the disease progresses to the metastatic stage. Slowly progressive or asymptomatic patients with small metastases present a different challenge from those with rapidly progressive, symptomatic disease, although in all cases the disease is not curable.

In asymptomatic patients, the goals of treatment are to control disease (i.e. to stop the tumour from growing, rather than trying to reduce tumour size) without exposing the patient to undue toxicity. In such patients, sequential single agents are the norm (if there is no appropriate clinical trial), and the choice depends on patient convenience, comorbidities and previous toxicities (95% of patients have already received neoadjuvant or adjuvant chemotherapy [29]). Possible treatments include taxanes, anthracyclines (e.g. liposomal doxorubicin), capecitabine, platinum agents, other microtubule-directed agents, vinorelbine and gemcitabine.

In patients with rapidly progressive and symptomatic metastatic disease, there is little need to balance efficacy and tolerability of treatments, because the disease is likely to cause more toxicity than therapy would. The goal of treatment is to achieve a tumour response, and combination regimens always have higher response rates than single agents. Options include combinations involving bevacizumab, docetaxel + capecitabine, paclitaxel + gemcitabine, and ixabepilone + capecitabine.

As with the neoadjuvant and adjuvant settings, the jury is still out on the benefit of platinum-based treatments in the metastatic setting. Cisplatin monotherapy achieved only a 10% response rate in largely treatment-naive patients [30]. Carboplatin monotherapy achieved a 17% response rate in patients who were largely pretreated with an EGFR inhibitor [31]. Until the results of further clinical trials are available, platinum-based treatment should probably not be a standard of care, although it could be considered in later lines of treatment for metastatic TNBC.

**Figure 3:**
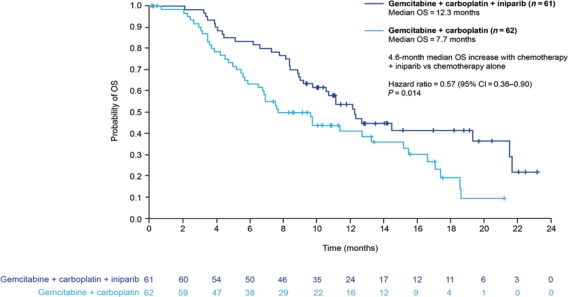
Overall survival in patients with triple-negative breast cancer treated with gemcitabine + carboplatin with or without iniparib (adapted from ref. [32])

Novel anticancer agents such as PARP inhibitors and iniparib are eliciting a great deal of interest currently, although none is yet available outside clinical trials. Most data are available for iniparib, which is provoking most excitement in the setting of sporadic (i.e. not *BRCA1*-associated) TNBC. Iniparib does not possess characteristics typical of the PARP inhibitor class and investigations are currently in progress to elucidate its main mechanism of action. The recently published phase II study demonstrated that the addition of iniparib to gemcitabine + carboplatin improved the clinical benefit and survival of patients with metastatic TNBC, compared with chemotherapy alone, without significantly increased toxic effects (Figure 3) [32]. However, in the pivotal phase III trial, iniparib demonstrated activity but did not meet the statistically rigorous primary endpoint [33], although it is possible that subsets within the larger trial will demonstrate benefit; those analyses are ongoing.

Questions that remain to be answered include: Is DNA damage stimulus needed in non-*BRCA*^+^ tumours? Might PARP inhibitors and iniparib work in any breast cancer or will the benefit be seen only in TNBC patients? What secondary effects might occur with prolonged prevention of DNA damage repair?

## Conclusions

In the opinions of this expert panel, three factors are critical when considering a patient with TNBC and deciding how best to manage her disease:

- The quality of the initial pathology
- The possibility of a genetic mutation, and the impact on the wider family
- The challenges posed by the heterogeneity of the disease and the range of treatment options available

Breast cancer mortality is decreasing [34], but most benefits are seen in patients with ER-positive or HER2-positive disease. The only way that treatment for TNBC can improve is through clinical trials of new agents and new strategies. Therefore all patients should be encouraged to participate in clinical trials.

## Figures and Tables

**Table 1:** Typical clinical and pathological features of triple-negative breast cancer (ref. [3,4]).

Clinical	Younger patients (47–55 years)
	African-American women
	Interval cancers
	BRCA1 mutations
Pathological features	High grade, high mitotic count
	Pushing borders (recalls medullary cancer of breast)
	Geographic necrosis, central fibrosis
	Stromal lymphocytic infiltrate
	Metaplasia
	Prevalence of brain and lung metastases even if the patient has negative lymph nodes

**Table 2:** Heterogeneity of tumour pathology and prognosis in triple-negative breast cancer.

**Tumour description**	**Prognosis**
Invasive ductal carcinoma not otherwise specified, high grade	Poor
Invasive lobular carcinoma, pleomorphic type, high grade	Poor
Metaplastic or myeloblastic carcinoma, high grade	Poor
High-grade oat-cell neuroendocrine tumours	Poor
Apocrine breast cancers (some may be HER2^+^)	Depends on grade: Grade 1 = good Grade 2 = intermediate Grade 3 = poor
Medullary	Good
Adenoid-cystic	Good
Metaplastic low-grade (low-grade adenosquamous, fibromatosis like)	Good

**Table 3:** Personal or familial features suggestive of hereditary cancer, as a guide for genetic testing (adapted from ASCO 2003 [26]).

Features suggestive of hereditary cancer among first-degree relatives (second-degree if paternal) Two women with breast cancer diagnosed before the age of 50 years One woman with breast cancer diagnosed before 50 years + one woman with ovarian cancer at any age *or* + one woman with bilateral breast cancer at any age Four women with breast cancer only One woman with breast + ovarian cancer One woman with breast cancer diagnosed before the age of 30 years One woman with triple-negative breast cancer diagnosed before 50 years
Offer genetic counselling before testing
Test affected family members first (i.e. those with history of breast cancer)
